# Unexpected Metastasis of Primary Colonic Adenocarcinoma: A Case Report and Literature Review

**DOI:** 10.7759/cureus.63299

**Published:** 2024-06-27

**Authors:** Meryem El jarroudi, Soufia El Ouardani, Jihane Derfoufi, Mohammed M Bakhti, Karich Nassira, Ouissam Al Jarroudi, Sami Aziz Brahmi, Amal Bennani, Said Afqir

**Affiliations:** 1 Medical Oncology, Mohammed VI University Hospital, Oujda, MAR; 2 Medical Oncology, Faculty of Medicine and Pharmacy of Oujda, Mohammed First University, Oujda, MAR; 3 Pathology, Mohammed VI University Hospital, Oujda, MAR; 4 Anatomopathology, Faculty of Medicine and Pharmacy of Oujda, Mohammed First University, Oujda, MAR

**Keywords:** colorectal cancer, hematuria, cystoscopy, bladder metastasis, transurethral resection

## Abstract

Colorectal cancer is a common cancer worldwide. The major sites of colorectal cancer metastasis are the liver, lungs, peritoneum, lymph nodes, and bones. However, secondary localization in the bladder is extremely rare. Herein, we present the case of a 36-year-old patient who underwent surgery for colonic adenocarcinoma. Subsequently, the patient presented total hematuria during adjuvant chemotherapy. Cystoscopy and biopsy identified a bladder metastasis. In our discussion, we aim to delve into the distinct characteristics of bladder metastases originating from digestive neoplasms.

## Introduction

Colorectal cancer is the third most common cancer worldwide, with at least 1.9 million new cases reported globally in 2020, accounting for 10.7% of new cases. Among women, there were 865,630 new cases, representing 9.9% [1]. Metastases of colorectal cancer occur through lymphatic, hematogenous, or direct spread. The most common secondary sites include the liver, lungs, peritoneum, lymph nodes, and bones [2]. However, bladder metastases can be discovered incidentally on radiological exams. Clinical differentiation between primary and secondary bladder tumors is difficult due to the similar symptoms [3]. The diagnosis confirmation is based on cystoscopy and biopsy [4]. Immunochemistry results should be interpreted carefully to rule out a primary bladder tumor. In this paper, we present the case of a 36-year-old patient who underwent surgery for colonic adenocarcinoma. Subsequently, the patient presented total hematuria during adjuvant chemotherapy. Cystoscopy and biopsy identified a bladder metastasis. In our discussion, we aim to delve into the distinct characteristics of bladder metastases originating from digestive neoplasms.

## Case presentation

**Figure 1:**
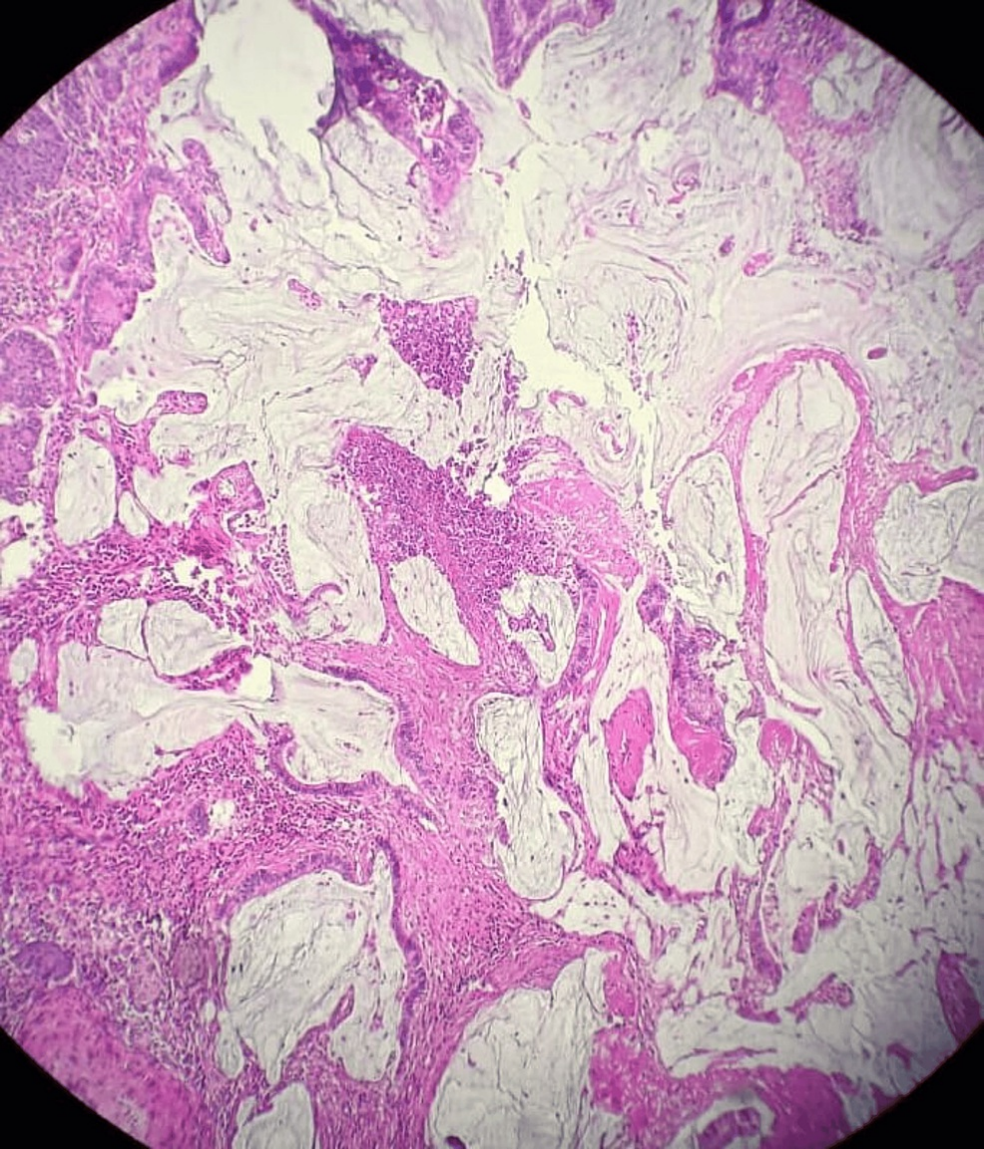
The tumor infiltrates the entire wall down to the subserosa

**Figure 2:**
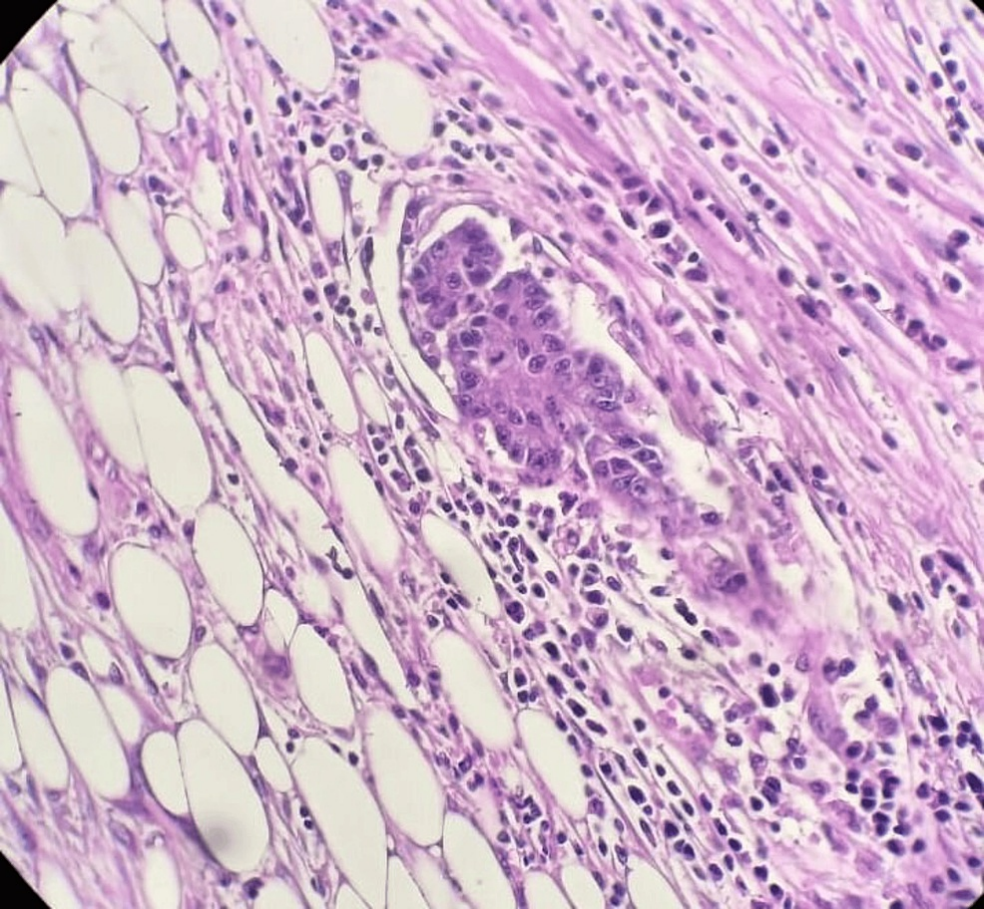
Tumor cells are irregular, anisokaryotic and hyperchromatic, with a mucinous background

**Figure 3:**
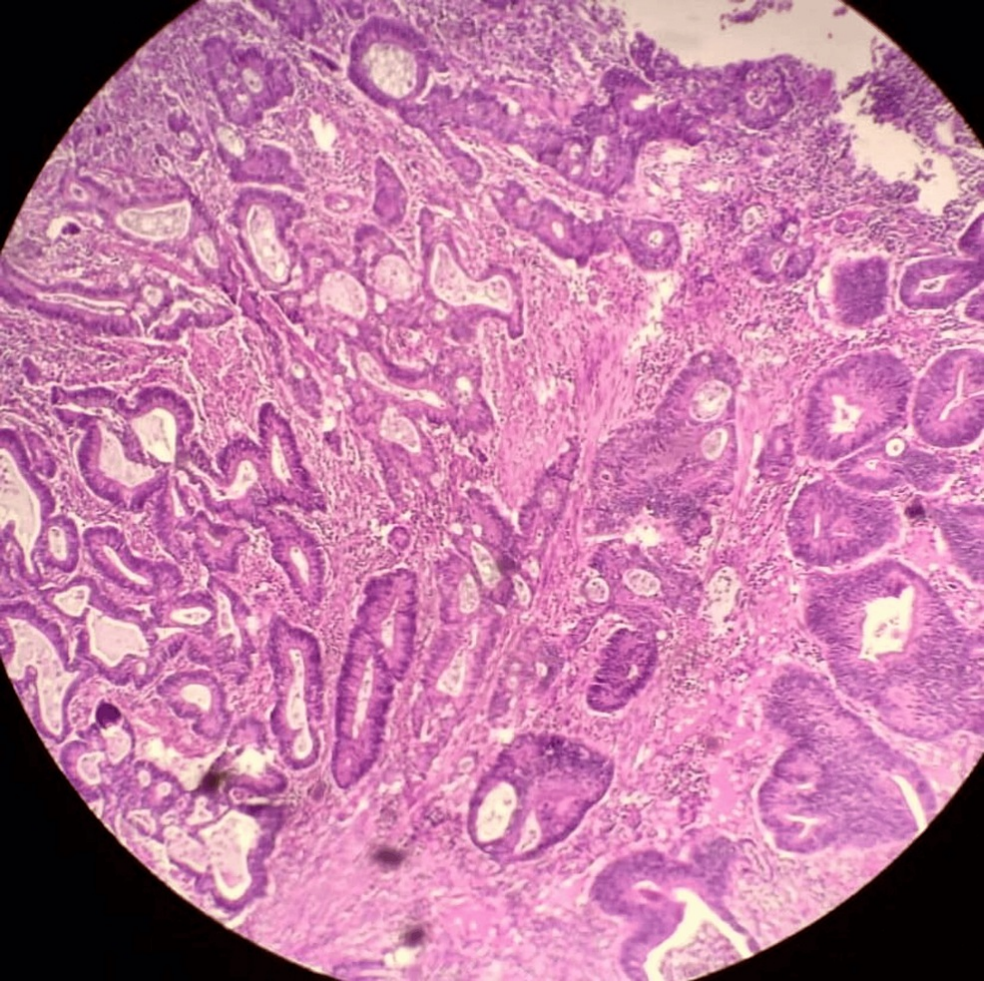
Carcinomatous proliferation of tubes and clumps

A 36-year-old patient, with a history of chronic anemia, was admitted due to abdominal pain accompanied by mild rectal bleeding. A CT scan of the abdomen and pelvis revealed thickening of the rectosigmoid wall with surrounding sclerolipomatosis phlegmons and extraintestinal air bubbles, suggestive of inflammatory bowel disease; however, a malignant origin couldn't be ruled out. Subsequent total colonoscopy identified an ulcerating, polypoid colonic tumor located 32 cm from the anal verge. A biopsy of the colon confirmed a well-differentiated ulcerating adenocarcinoma infiltrating the colonic mucosa (Figures 1-3).

**Figure 4:**
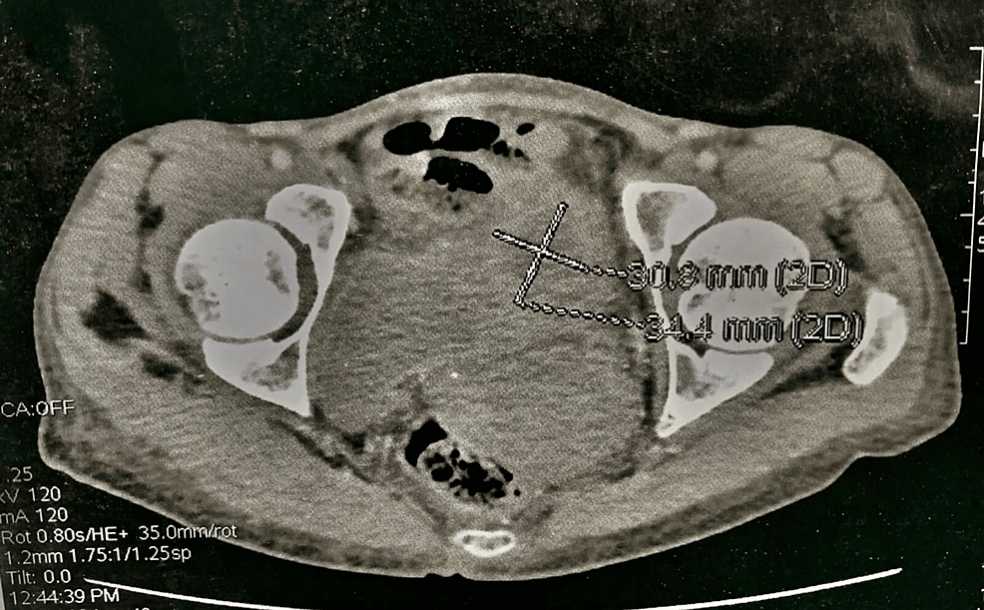
CT scan demonstrated a bladder mass situated at the dome

The patient underwent colonic resection with typical end-to-end anastomosis. Subtotal hysterectomy was performed on suspicion of tumor infiltration. Histopathological analysis revealed a mucinous adenocarcinoma reaching the subserosa, with clear surgical margins, perineural sheath involvement, but no vascular emboli. Lymph node dissection detected one positive node out of 16, with no evidence of hepatic infiltration. The tumor was staged as PT3N1M0. Subsequently, the patient received adjuvant chemotherapy with FOLFOX but developed total hematuria after one cycle. A follow-up CT scan demonstrated a bladder mass situated at the dome, protruding into the lumen, adherent to the small bowel loops, and the wall of the low-lying cecum (Figure 4).

**Figure 5:**
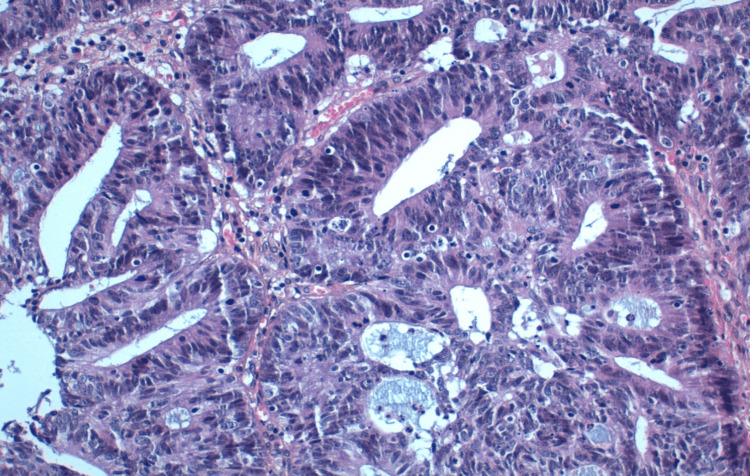
At high x20 magnification, tumor proliferation, with cells displaying obvious cytonuclear atypia and multi-layered nuclei

**Figure 6:**
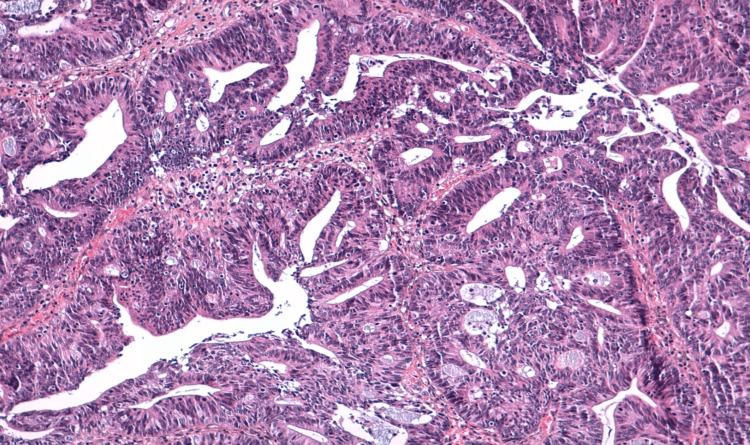
At low x10 magnification, this tumor proliferation is essentially arranged in cribriform masses and glands, resting on a fibro-inflammatory stroma

**Figure 7:**
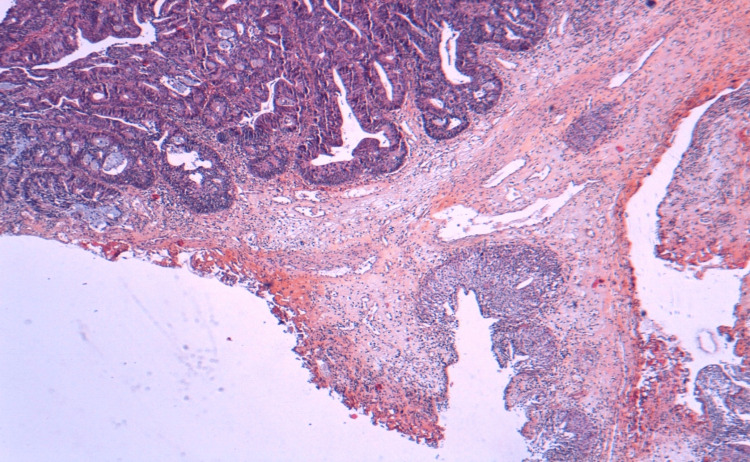
At low magnification x4, the bladder mucosa is the site of malignant tumor proliferation

Transurethral resection of the bladder confirmed moderately differentiated intestinal-type adenocarcinoma involving the bladder (Figures 5-7).

The decision of the multidisciplinary consultation meeting was to continue the treatment with FOLFOX and discussion of surgical treatment based on response.

## Discussion

Bladder metastases are a rare occurrence, comprising only 2% of all malignant bladder tumors observed in surgical specimens. Extension can occur either contiguously or from distant organs, with distant metastases from a primary colorectal tumor accounting for 21% [5].

Bates et al. analyzed 282 secondary bladder neoplasms, representing 2.3% of all malignant bladder tumors in surgical specimens. The most common primary sites were the colon 21% of secondary neoplasms), prostate (19%), rectum (12%), and cervix (11%) [5].

Sheehan et al. found bladder metastases in 0.4% of autopsies involving 2500 patients. The primary sites were the skin in eight cases, the stomach in six cases, the lung in five cases, the pancreas in one case, and the colon in one case [6].

The main symptoms prompting consultation are hematuria, dysuria, and sometimes bladder metastases are incidentally discovered on radiological examination [3]. Acute abdominal pain can be a telltale sign of bladder metastasis [7]. Confirmation of the diagnosis is based on cystoscopy with biopsy [4]. It is often difficult to distinguish primary adenocarcinomas from secondary colonic adenocarcinomas in the bladder because they appear morphologically similar [8].

Wang and al demonstrated that the immunohistochemical panel including CK7, CK20, TM, and β-catenin are sufficient to exclude a primary bladder adenocarcinoma [9]. In another study, Raspollini proved that immunostaining with antibodies anti-CDX-2, CK7, CK20, and CEA can be used to differentiate a primary bladder adenocarcinoma from a secondary colorectal adenocarcinoma [8].

There are few data available in the literature on the management and survival of secondary bladder neoplasms, most of which are early studies or small series [10,11].

The treatment of bladder metastasis depends on many factors: performance status, presence of other metastases, the primary tumor origin, symptoms, and treatment received. In our presentation, the patient underwent a FOLFOX regimen as a first-line chemotherapy for metastatic colonic cancer with bladder metastasis.

In recent years, significant progress has been made in the treatment of metastatic colorectal cancer, surpassing the mere use of chemotherapy as the standard treatment. Targeted therapies and immunotherapy have emerged as crucial alternatives, thereby avoiding the adverse effects of cytotoxicity and reducing the development of chemotherapy resistance [10].

Targeted therapy has significantly improved overall patient survival [11]. Additionally, Immunotherapy with agents such as pembrolizumab and nivolumab has demonstrated efficacy in patients with metastatic colorectal cancer and a deficiency in mismatch repair and high microsatellite instability (dMMR-MSI-H) [12].

Regarding the diagnosis and treatment of bladder tumors, transurethral bladder resection provides both diagnostic and therapeutic opportunities. Concurrently, bladder irrigation with tranexamic acid has proven effective in emergency situations [13].

## Conclusions

Although secondary bladder tumors remain rare, hematuria is the most common symptom. The diagnosis is based on cystoscopy with biopsy and immunohistochemical study to exclude a primary bladder tumor. The prognosis of bladder metastasis is mainly related to the stage of the primary tumor and the response to treatment. Generally, the presence of bladder metastasis is associated with a poor prognosis and a major impairment of quality of life.
